# Porcine Reproductive and Respiratory Syndrome Virus Activates Lipophagy To Facilitate Viral Replication through Downregulation of NDRG1 Expression

**DOI:** 10.1128/JVI.00526-19

**Published:** 2019-08-13

**Authors:** Jiang Wang, Jiao-Yang Liu, Ke-Yu Shao, Ying-Qian Han, Guo-Li Li, Sheng-Li Ming, Bing-Qian Su, Yong-Kun Du, Zhong-Hu Liu, Gai-Ping Zhang, Guo-Yu Yang, Bei-Bei Chu

**Affiliations:** a College of Animal Sciences and Veterinary Medicine, Henan Agricultural University, Zhengzhou, Henan Province, People’s Republic of China; University of Kentucky College of Medicine

**Keywords:** autophagy, lipid droplet, lipophagy, N-Myc downstream-regulated gene 1, porcine reproductive and respiratory syndrome virus

## Abstract

Autophagy maintains cellular homeostasis by degrading organelles, proteins, and lipids in lysosomes. Autophagy is involved in the innate and adaptive immune responses to a variety of pathogens. Some viruses can hijack host autophagy to enhance their replication. However, the role of autophagy in porcine reproductive and respiratory syndrome virus (PRRSV) infection is unclear. Here, we show that N-Myc downstream-regulated gene 1 (*NDRG1*) deficiency induced autophagy, which facilitated PRRSV replication by regulating lipid metabolism. NDRG1 mRNA is expressed ubiquitously in most porcine tissues and most strongly in white adipose tissue. PRRSV infection downregulated the expression of NDRG1 mRNA and protein, while NDRG1 deficiency contributed to PRRSV RNA replication and progeny virus assembly. NDRG1 deficiency reduced the number of intracellular lipid droplets (LDs), but the expression levels of key genes in lipogenesis and lipolysis were not altered. Our results also show that NDRG1 deficiency promoted autophagy and increased the subsequent yields of hydrolyzed free fatty acids (FFAs). The reduced LD numbers, increased FFA levels, and enhanced PRRSV replication were abrogated in the presence of an autophagy inhibitor. Overall, our findings suggest that NDRG1 plays a negative role in PRRSV replication by suppressing autophagy and LD degradation.

**IMPORTANCE** Porcine reproductive and respiratory syndrome virus (PRRSV), an enveloped single-positive-stranded RNA virus, causes acute respiratory distress in piglets and reproductive failure in sows. It has led to tremendous economic losses in the swine industry worldwide since it was first documented in the late 1980s. Vaccination is currently the major strategy used to control the disease. However, conventional vaccines and other strategies do not provide satisfactory or sustainable prevention. Therefore, safe and effective strategies to control PRRSV are urgently required. The significance of our research is that we demonstrate a previously unreported relationship between PRRSV, NDRG1, and lipophagy in the context of viral infection. Furthermore, our data point to a new role for NDRG1 in autophagy and lipid metabolism. Thus, NDRG1 and lipophagy will have significant implications for understanding PRRSV pathogenesis for developing new therapeutics.

## Abstract (precis)

Porcine reproductive and respiratory syndrome virus (PRRSV), an enveloped single-positive-stranded RNA virus, causes acute respiratory distress in piglets and reproductive failure in sows. It has led to tremendous economic losses in the swine industry worldwide since it was first documented in the late 1980s. Vaccination is currently the major strategy used to control the disease. However, conventional vaccines and other strategies do not provide satisfactory or sustainable prevention. Therefore, safe and effective strategies to control PRRSV are urgently required. The significance of our research is that we demonstrate a previously unreported relationship between PRRSV, NDRG1, and lipophagy in the context of viral infection. Furthermore, our data point to a new role for NDRG1 in autophagy and lipid metabolism. Thus, NDRG1 and lipophagy will have significant implications for understanding PRRSV pathogenesis for developing new therapeutics.

## INTRODUCTION

Porcine reproductive and respiratory syndrome (PRRS) is a highly contagious swine disease that causes acute respiratory distress in piglets and reproductive failure in sows. It has led to tremendous economic losses in the swine industry worldwide since it was first documented in the late 1980s (1, 2). The etiological agent of this disease, porcine reproductive and respiratory syndrome virus (PRRSV), is an enveloped single-positive-stranded RNA virus of the family *Arteriviridae* (3). In recent years, outbreaks of highly virulent variants of the virus in China have led to concerns within the global swine industry (4). Vaccination is currently the major strategy used to control the disease. However, conventional vaccines and other strategies do not provide satisfactory or sustainable prevention (5). Therefore, safe and effective strategies to control PRRSV are urgently required.

PRRSV relies on host factors to complete its replication cycle. The first contact between the virus and macrophages usually occurs via the heparan sulfate on the cell surface (6). This weak interaction is then strengthened by the interaction between the sialoadhesin molecule on macrophages and the viral GP5/M heterodimer (7). This is followed by the uptake of the virus-receptor complex via clathrin-mediated endocytosis. Upon its internalization, the viral genome is released into the cytoplasm, which requires host CD163 and cathepsin E (8). Moreover, recent studies have found that CD163 is indispensable for facilitating PRRSV infection both *in vitro* and *in vivo* (9). Antibodies recognizing CD163-SRCR5 (scavenger receptor cysteine-rich domain 5) can block PRRSV infection *in vitro*, thus suggesting that SRCR5 is essential for viral infection. Moreover, the soluble form of truncated CD163-SRCR5-SRCR9 can bind PRRSV particles and inhibit virus proliferation *in vitro* (10). CD163^−/−^ pigs are resistant to PRRSV infection, thus indicating that CD163 is the most important receptor (11). Once inside the cytoplasm, the linear viral plus-strand RNA genome undergoes translation, transcription, and replication by hijacking the host translational and transcriptional machinery (12). The preformed nucleocapsid then buds through the smooth endoplasmic reticulum (ER)/Golgi complex to produce enveloped viral particles. These viral particles finally accumulate in intracellular vesicles, which are released from the cell by exocytosis. Although some cellular and viral factors have been implicated in these processes (13–15), their precise functions and modes of action remain unknown. Further investigation of these host-virus interactions is essential to the development of new therapeutic strategies against PRRSV.

N-Myc downstream-regulated gene 1 (*NDRG1*) is a member of the N-Myc downregulated gene family, which belongs to the alpha/beta hydrolase superfamily. The NDRG1 protein is highly conserved among species and is ubiquitously expressed in most human tissues (16). It participates in diverse cellular functions, including stress responses (17), differentiation (18), cell proliferation (19), cell cycle regulation (20), vesicular trafficking (21), metastasis suppression, and p53-mediated apoptosis (22). Recent studies have demonstrated that NDRG1 regulates the NF-κB (23), transforming growth factor β (TGF-β) (24), WNT-β (25), RAS/RAF/MEK/extracellular signal-regulated kinase (ERK) (16), and phosphatidylinositol 3-kinase (PI3K)/AKT/MTOR (26) signaling pathways. NDRG1 is also thought to regulate autophagy. The upregulation of NDRG1 initiates BNIP3- and Beclin-mediated autophagy (27) and acts as an autophagy inhibitor during ER stress (28). Therefore, NDRG1 may play a role in tuning autophagy levels by acting on different autophagy-inducing or -inhibiting pathways. The conflicting results in different studies may be attributable to the use of different tissues or cell types. Recently, NDRG1 has been shown to restrict the propagation of hepatitis C virus (HCV) by inhibiting viral assembly (29). On the contrary, Chen et al. found that NDRG1 facilitates influenza A virus (IAV) replication by suppressing canonical NF-κB signaling (30). However, the role of NDRG1 in the PRRSV life cycle requires further investigation.

Autophagy is a cellular process by which intracellular proteins and organelles are degraded in the lysosomes to supply the cell with energy and to maintain cellular homeostasis (31, 32). Cells store fat in the form of lipid droplets (LDs), intracellular deposits of lipid esters surrounded by a monolayer of phospholipids and separated from the hydrophilic cytosolic environment by a coat of structural proteins (33, 34). Autophagy has been linked to the lipolysis of LDs because it fuses with lysosomes (lipophagy) (35, 36). The hydrolysis of triglycerides (TGs) inside LDs into free fatty acids (FFAs) through lipophagy drives mitochondrial β-oxidation, which supplies cells with ATP (37). In addition to lipophagy, autophagy is also closely related to viral infection. Autophagy contributes to the cell’s defense against Sindbis virus (38), herpes simplex virus 1 (39), and vesicular stomatitis virus (40) infections. However, some viruses have evolved a means to subvert the host autophagic response to ensure their survival or replication (41). Dengue virus (DENV) (37), poliovirus (42), HCV (43), and coxsackievirus B3 (44), have been shown to require cellular autophagy for their efficient replication. Markers of poliovirus replication colocalize with autophagosomes in infected cells (45). Furthermore, studies have shown that PRRSV utilizes autophagy to facilitate viral replication. Sun et al. have found that PRRSV induces incomplete autophagy, and this inhibition of autophagosome and lysosome fusion leads to an accumulation of autophagosomes, which may serve as replication sites that enhance PRRSV replication (46). Liu et al. have demonstrated that induction of autophagy by rapamycin significantly enhances the viral titers of PRRSV. However, they have also shown that functional autolysosomes can form after PRRSV infection, and an autophagosome-lysosome fusion inhibitor decreases viral titers, thus suggesting that PRRSV induces complete autophagy (47). Regarding the mechanism through which autophagy participates in viral replication, Zhou et al. have shown that autophagy can postpone PRRSV-induced apoptosis in MARC-145 cells through Bad-Beclin1 interactions (48). More investigations must be conducted to provide additional evidence regarding the specific mechanisms of autophagy involved in PRRSV proliferation.

Here, we examined the contribution of autophagy to lipid catabolism and the consequences of this autophagic function for PRRSV replication. We demonstrate that PRRSV infection reduces NDRG1 expression, which leads to the autophagy-dependent processing of LDs and the release of FFAs. Our results demonstrate the role of autophagy in PRRSV infection and provide a mechanism in which the virus adapts cellular lipid metabolism to promote its replication.

## RESULTS

### Expression profile of porcine NDRG1.

**FIG 1:**
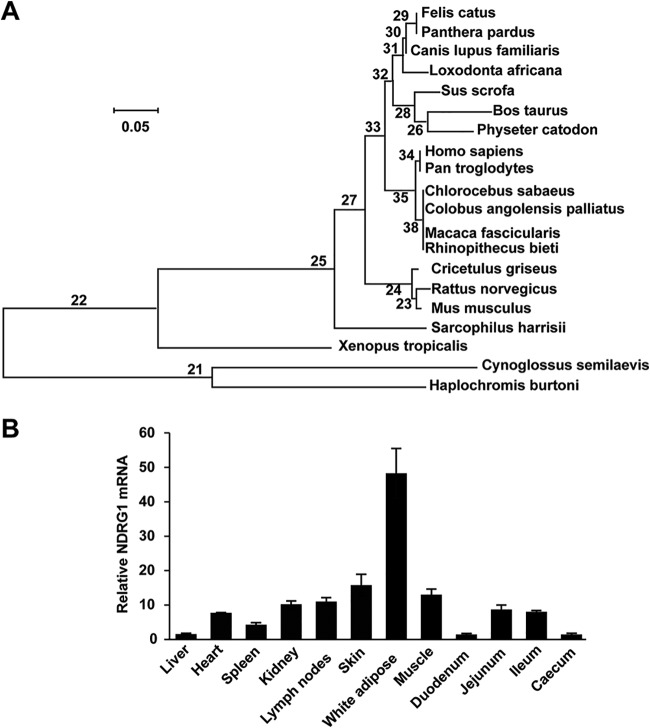
Expression analysis of NDRG1. (A) Phylogenetic tree of NDRG1 proteins constructed with MEGA6 software. Protein sequences of NDRG1 from different species were taken from GenBank, under accession numbers XP_020944534 (Sus scrofa), NP_001030181 (Bos taurus), NP_001011991 (Rattus norvegicus), NP_032707 (Mus musculus), NP_001128714 (Homo sapiens), XP_007999783 (*Chlorocebus sabaeus*), XP_005628031 (Canis lupus familiaris), XP_012820024 (Xenopus tropicalis), XP_019315342 (Panthera pardus), XP_017730622 (Rhinopithecus bieti), XP_012397285 (Sarcophilus harrisii), XP_005935015 (Haplochromis burtoni), XP_005564176 (Macaca fascicularis), XP_011793534 (Colobus angolensis *palliatus*), XP_008321638 (Cynoglossus semilaevis), XP_007106495 (Physeter catodon), XP_023104387 (Felis catus), XP_010586814 (Loxodonta africana), XP_009454233 (Pan troglodytes), and XP_007605917 (Cricetulus griseus). (B) *NDRG1* mRNA levels were detected in porcine tissues by RT-qPCR. Values were normalized to the β-actin (*ACTB*) mRNA levels. Relative amounts of *NDRG1* mRNA were compared with those in the liver. Data represent means ± standard errors of the means from three independent experiments.

To characterize the role of NDRG1 in PRRSV replication, we first searched for the predicted coding DNA sequence (CDS) of NDRG1 (GenBank accession number XP_020944534) in the National Center for Biotechnology Information (NCBI) database. Porcine NDRG1 contains 384 amino acid residues and shares 96%, 92%, and 93% sequence identity at the amino acid level with its bovine, mouse, and human orthologues, respectively. A sequence alignment of the NDRG1 phosphorylation sites showed that the phosphorylation sites at Ser330 and Thr346, which are reportedly important for NDRG1 activity (29), are highly conserved in the human, mouse, bovine, and porcine genomes (data not shown). In a phylogenetic analysis, porcine, bovine, and sperm whale NDRG1 clustered together (Fig. 1A). A semiquantitative reverse transcription-PCR (RT-PCR) analysis indicated that *NDRG1* was ubiquitously expressed in all the pig tissues examined, and the highest level of *NDRG1* mRNA was detected in white adipose tissue (Fig. 1B). However, previous studies have shown that although *NDRG1* mRNA is ubiquitously expressed in most human tissues, it is expressed most strongly in epithelial cells (49, 50), suggesting that the function of NDRG1 differs among species. Taken together, our data suggest that porcine NDRG1 may have a specific function related to fatty acid (FA) metabolism in white adipose tissue.

### PRRSV infection downregulates NDRG1 expression.

**FIG 2:**
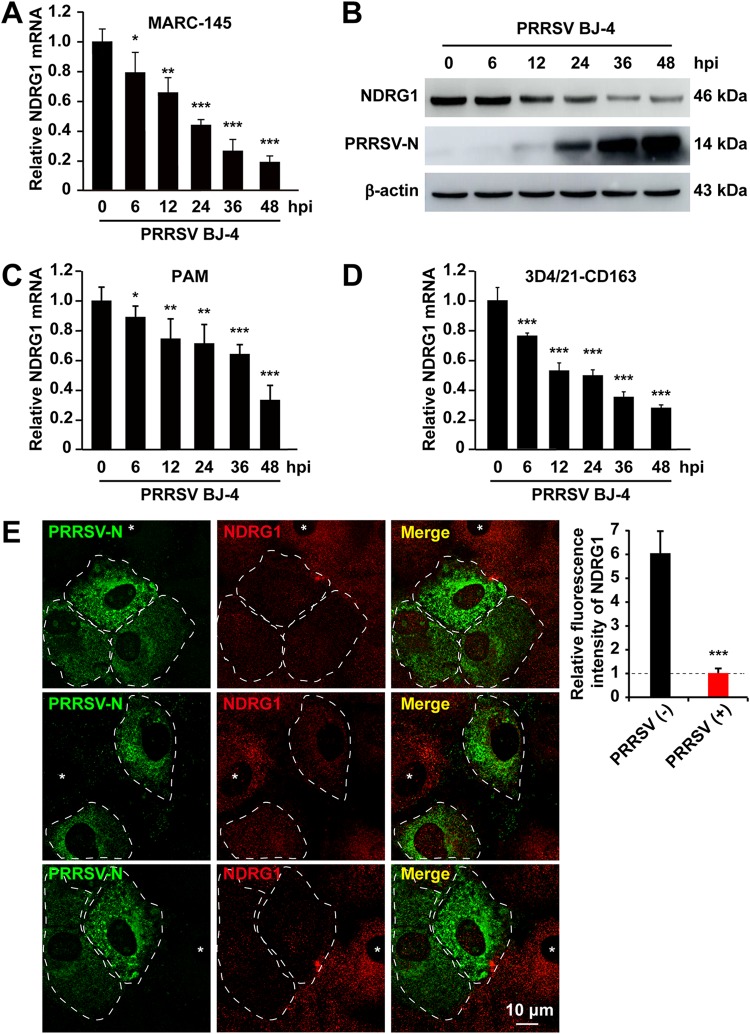
PRRSV infection reduces NDRG1 mRNA and protein levels. (A) MARC-145 cells were infected with PRRSV BJ-4 at an MOI of 10 for the indicated times. RNA was extracted from uninfected and infected cells. *NDRG1* mRNA levels were detected by RT-qPCR. Values were normalized to β-actin (*ACTB*) mRNA levels. Data are the means ± standard errors of the means from three independent experiments. *, *P < *0.05; **, *P < *0.01; ***, *P < *0.0001 (by one-way ANOVA). hpi, hours postinfection. (B) Lysates of uninfected or infected cells were analyzed by immunoblotting using anti-NDRG1 and anti-PRRSV-N antibodies. β-Actin was used as the loading control. (C) PAM cells were infected with PRRSV BJ-4 at an MOI of 5 for the indicated times. RNA was extracted from uninfected and infected cells. *NDRG1* mRNA levels were detected by RT-qPCR. (D) 3D4/21-CD163 cells were infected with PRRSV BJ-4 at an MOI of 10 for the indicated times. RNA was extracted from uninfected and infected cells. *NDRG1* mRNA levels were detected by RT-qPCR. (E) Staining for NDRG1 was reduced in PRRSV-infected cells. MARC-145 cells were infected with PRRSV BJ-4 at an MOI of 10 for 48 h and then fixed and stained with antibodies directed against PRRSV-N (green) and NDRG1 (red). White dotted lines highlight infected cells, and asterisks indicate uninfected cells. Fluorescence intensities were quantified in both uninfected and PRRSV-positive cells (*n* = 40) with ImageJ. ***, *P < *0.0001 (by an unpaired two-tailed *t* test).

**FIG 3:**
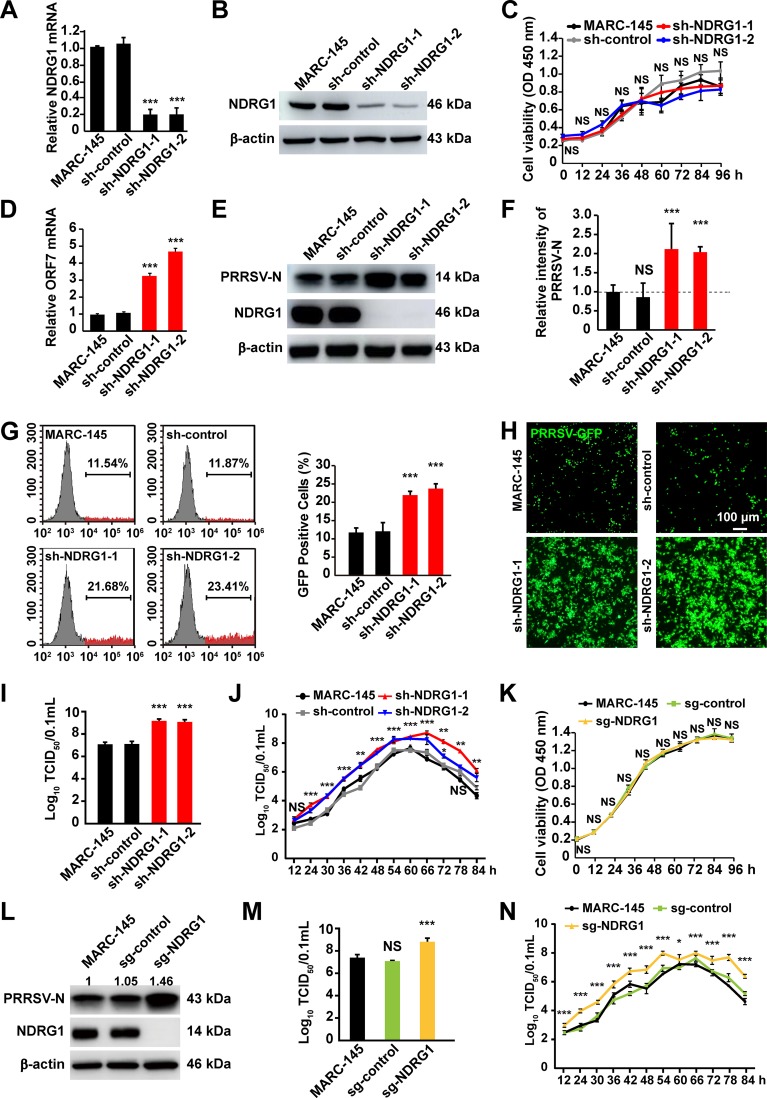
Knockdown of NDRG1 promotes PRRSV infection. (A) *NDRG1* mRNA levels in cells stably expressing shRNAs were detected by RT-qPCR. MARC-145 cells and scrambled control shRNA (sh-control)-expressing cells were used as controls. Values were normalized to the β-actin (*ACTB*) mRNA levels. ***, *P < *0.0001 (by one-way ANOVA). (B) Immunoblot analysis of NDRG1 expression in cells from panel A. Antibodies used are indicated on the left. (C) Proliferation of MARC-145 cells stably expressing sh-control or sh-NDRG1 was determined by a CCK assay. OD, optical density; NS, not significant (by one-way ANOVA). (D) Cells were infected with PRRSV BJ-4 (MOI = 10) for 48 h. The mRNA levels of PRRSV ORF7 in the cells were detected by RT-qPCR. ***, *P < *0.0001 (by one-way ANOVA). (E) Cells were infected with PRRSV BJ-4 (MOI = 10) for 48 h. Immunoblot analyses were performed with the indicated antibodies. (F) Semiquantitative densitometric analysis of PRRSV-N from panel E was performed with ImageJ. Protein content was normalized to the corresponding β-actin content. NS, not significant; ***, *P* < 0.0001 (by one-way ANOVA). (G) Cells were infected with PRRSV-GFP (MOI = 10) for 48 h, and fluorescence-positive cells were measured by flow cytometry. Gray peaks, GFP-negative cells (cells not infected with PRRSV); red peaks, GFP-positive cells (cells infected with PRRSV). ***, *P* < 0.0001 (by one-way ANOVA). (H) Cells were infected with PRRSV-GFP (MOI = 10) for 48 h, and fluorescence was detected with a fluorescence microscope. The bottom panel shows bright-field images of the corresponding cells. (I) Cells were infected with PRRSV BJ-4 (MOI = 10) for 48 h. Virus was harvested with three freeze-thaw cycles, and the viral titer was determined by a TCID_50_ assay. ***, *P < *0.0001 (by one-way ANOVA). (J) Growth curve of PRRSV BJ-4. Cells were incubated with PRRSV BJ-4 (MOI = 10) at 4°C for 1 h and then washed twice with PBS. At each hour after infection, the infected cells were frozen and thawed twice in an equal volume of the supernatant for titrating the intracellular virus with the TCID_50_ assay. (K) Proliferation of MARC-145 cells and MARC-145 cells transfected with control sgRNA (sg-control) or with sgRNA mediating NDRG1 knockout (sg-NDRG1) was determined by a CCK assay. NS, not significant (by one-way ANOVA). (L) Cells were infected with PRRSV BJ-4 (MOI = 10) for 48 h. Immunoblot analyses were performed with the indicated antibodies. (M) Growth curve of PRRSV BJ-4 in NDRG1^−/−^ cells. Cells were incubated with PRRSV BJ-4 (MOI = 10) at 4°C for 1 h and then washed twice with PBS. At each hour after infection, the infected cells were frozen and thawed twice in an equal volume of the supernatant for titrating the intracellular virus by a TCID_50_ assay. (N) Cells were infected with PRRSV BJ-4 (MOI = 10) for 48 h. Virus was harvested with three freeze-thaw cycles, and the viral titer was determined by a TCID_50_ assay. ***, *P < *0.0001 (by one-way ANOVA).

To evaluate the expression levels of NDRG1 in response to PRRSV infection, MARC-145 cells were infected with PRRSV BJ-4 for 6, 12, 24, 36, or 48 h, and cells were then processed to measure the NDRG1 mRNA and protein levels. As shown in Fig. 2A, PRRSV infection caused a gradual reduction in *NDRG1* mRNA levels compared with those in the uninfected control group. Consistent with the mRNA levels, NDRG1 protein expression, detected with an NDRG1-specific antibody, was also downregulated (Fig. 2B). PRRSV-infected cells displayed strongly reduced NDRG1 levels at 12 h postinfection, the PRRSV N protein (PRRSV-N) was detectable simultaneously at 12 h postinfection, and its levels peaked at 48 h, which correlated with the observed decline in the NDRG1 levels. Consistently, we observed the same effect, in which PRRSV infection suppressed the expression of NDRG1 on porcine alveolar macrophages (PAMs) (Fig. 2C) and 3D4/21 cells expressing CD163 (Fig. 2D). We confirmed this phenotype by immunofluorescence using confocal microscopy and observed a substantial reduction in NDRG1 staining in cells positive for the PRRSV N protein (Fig. 2E, white dotted lines) relative to that in adjacent uninfected cells (Fig. 2E, asterisks). Quantification of the mean fluorescence intensity revealed a significant reduction in NDRG1 in the N-protein-positive cells. Taken together, these data indicate that PRRSV infection suppressed the expression of NDRG1.

### Knockdown of NDRG1 increases PRRSV replication.

To determine the role of NDRG1 in PRRSV replication, lentivirus-delivered short hairpin RNAs (shRNAs) were used to stably knock down NDRG1. All the cells transfected with shRNA targeting NDRG1 (sh-NDRG1) displayed significant reductions in their *NDRG1* mRNA levels compared with MARC-145 and control shRNA (sh-control)-transfected cells (Fig. 3A). The knockdown efficiency was confirmed by immunoblotting, which indicated a 60% to 70% reduction in the NDRG1 protein levels (Fig. 3B). Cell viability, measured using a cell counting kit (CCK) assay, demonstrated that the knockdown of NDRG1 had no effect on the growth of MARC-145 cells (Fig. 3C). These cells were then infected with PRRSV BJ-4 for 48 h. To examine PRRSV replication, the viral RNA was isolated and quantified by RT-quantitative PCR (qPCR). We confirmed that NDRG1 knockdown significantly increased the mRNA levels of open reading frame 7 (ORF7) in sh-NDRG1 cells compared with the levels in control cells (Fig. 3D). Furthermore, the amounts of viral N protein production clearly increased at 48 h postinfection (Fig. 3E and F). Flow cytometry was used to analyze the replication of PRRSV-GFP (green fluorescent protein). As shown in Fig. 3G, the proportion of GFP-positive cells (red peaks) was significantly greater in the NDRG1 knockdown groups (21.68% and 23.41%) than in the control groups (11.54% and 11.87%, respectively; *P < *0.0001). Fluorescence microscopy also showed more pronounced PRRSV-mediated GFP expression in the sh-NDRG1 cells (Fig. 3H). The increase in PRRSV production was confirmed by measuring the viral titers in cells expressing sh-NDRG1 (Fig. 3I). Next, we determined the growth curves of PRRSV BJ-4 in MARC-145 and NDRG1 knockdown cells. The growth rate and titer of PRRSV in NDRG1 knockdown cells were higher than those in control cells (Fig. 3J). To further confirm these phenotypes, we used CRISPR/Cas9 technology to specifically target exon 4 of the NDRG1 gene in MARC-145 cells. A cell viability assay showed that NDRG1 knockout did not affect the growth of MARC-145 cells (Fig. 3K), and the results demonstrated that the expression of PRRSV-N (Fig. 3L) and the titer (Fig. 3M and N) were greatly increased in NDRG1^−/−^ (Cas9/NDRG1 single guide RNA [sgRNA] [sg-NDRG1]) cells. Together, these findings suggest that NDRG1 plays a negative role in PRRSV replication.

### NDRG1 overexpression inhibits PRRSV replication.

**FIG 4:**
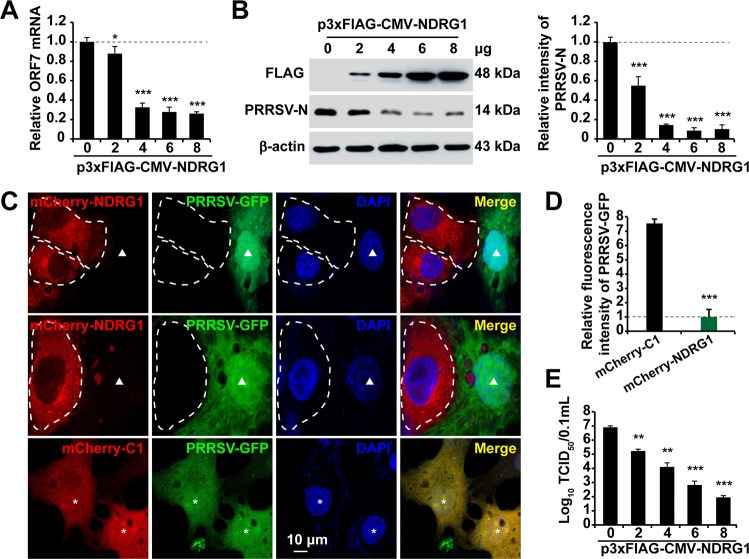
Overexpression of NDRG1 reduces PRRSV replication. (A) MARC-145 cells were transfected with different concentrations of a plasmid encoding FLAG-NDRG1 or with p3×Flag-CMV-10 for 24 h and then infected with PRRSV BJ-4 (MOI = 10) for 36 h. The mRNA levels of PRRSV ORF7 were detected by RT-qPCR. Values were normalized to the β-actin (*ACTB*) mRNA levels. *, *P < *0.05; ***, *P* < 0.0001. (B) Immunoblot analysis of PRRSV-N expression in cells from panel A. Antibodies used are indicated on the left. β-Actin was used as the loading control. Semiquantitative densitometric analyses of FLAG-NDRG1 and PRRSV-N were performed using ImageJ software. The protein content was normalized to the corresponding β-actin level. ***, *P* < 0.0001 (by one-way ANOVA). (C) MARC-145 cells were transfected with plasmid mCherry-NDRG1 or mCherry-C1 for 24 h and then infected with PRRSV-GFP at an MOI of 10 for 36 h. Cells were fixed and stained with DAPI (4′,6-diamidino-2-phenylindole), and their fluorescence was detected by fluorescence microscopy. White dotted lines highlight NDRG1-transfected cells, arrowheads indicate PRRSV-GFP-infected cells transfected with mCherry-NDRG1, and asterisks indicate PRRSV-GFP-infected cells transfected with pmCherry-C1. (D) GFP fluorescence intensities were quantified in cells transfected with both pmCherry-C1 and pmCherry-NDRG1 (*n* = 40) using ImageJ. Data are means ± standard errors of the means. ***, *P < *0.0001 (by an unpaired two-tailed *t* test). (E) MARC-145 cells were transfected with different concentrations of a plasmid encoding FLAG-NDRG1 or with p3×Flag-CMV-10 for 24 h and then infected with PRRSV BJ-4 (MOI = 10) for 36 h. Virus was harvested with three freeze-thaw cycles, and the viral titer was determined by a TCID_50_ assay. **, *P < *0.01; ***, *P < *0.0001 (by one-way ANOVA).

The results described above suggest that NDRG1 restricts PRRSV replication. To confirm the negative role of NDRG1 in PRRSV replication, MARC-145 cells were transfected with various concentrations of a plasmid encoding FLAG-NDRG1 or the empty vector for 24 h and then infected with PRRSV BJ-4. RT-qPCR and immunoblot analysis indicated that the increase in NDRG1 protein levels abrogated the amounts of PRRSV mRNA (Fig. 4A) and protein (Fig. 4B) produced. We also confirmed this phenotype by fluorescence confocal microscopy and observed substantial reductions in PRRSV-mediated GFP expression in cells positive for mCherry-NDRG1 (Fig. 4C, white dotted lines) compared with that in the adjacent uninfected cells (Fig. 4C, arrowheads). Cells transfected with the empty mCherry-C1 vector had no effect on PRRSV replication (Fig. 4C, asterisks). Quantification of the mean fluorescence intensity revealed a significant reduction in the GFP signal in the mCherry-NDRG1-positive cells (*n* = 40) (Fig. 4D). The titer assay also indicated that overexpression of NDRG1 reduced the viral titer and exhibited a dose-dependent effect (Fig. 4E). Together, these results highlight the negative role of NDRG1 in PRRSV replication.

### NDRG1 restricts PRRSV RNA replication and viral assembly.

**FIG 5:**
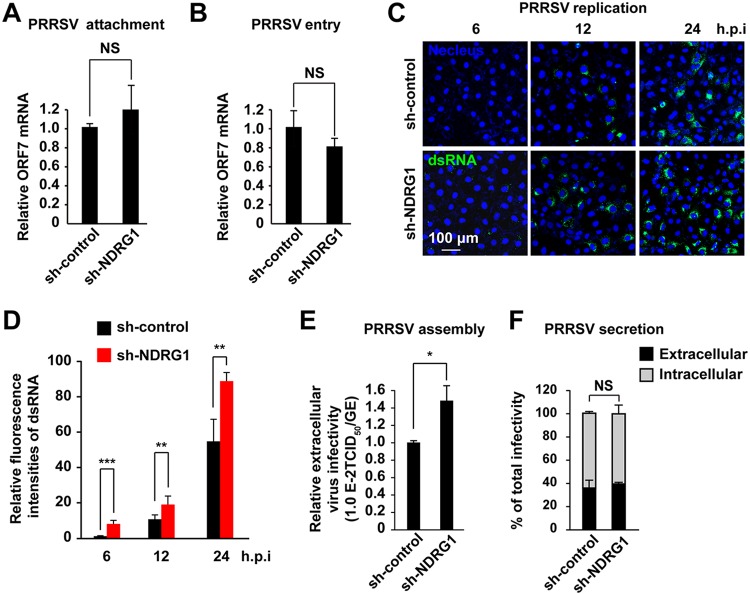
NDRG1 knockdown increases PRRSV replication and assembly. (A) PRRSV BJ-4 (MOI = 10) was allowed to bind to the surfaces of sh-control and sh-NDRG1 cells on ice for 1 h. After the cells were washed with PBS, the viral RNA was isolated and quantified by RT-qPCR with ORF7-specific primers. NS, not significant (by an unpaired two-tailed *t* test). (B) PRRSV BJ-4 (MOI = 10) was allowed to bind to the surfaces of sh-control and sh-NDRG1 cells on ice for 1 h (binding), and cells were then shifted to 37°C for 2 h (internalization). Intracellular viral RNA was isolated and quantified by RT-qPCR using ORF7-specific primers. NS, not significant (by an unpaired two-tailed *t* test). (C) Cells were infected with PRRSV BJ-4 at an MOI of 10 for the indicated times, before the cells were fixed and stained for double-stranded RNA (dsRNA) and with DAPI. Random fields of view were recorded with a confocal microscope. (D) Fluorescence intensities of dsRNA in panel C were quantified in cells containing PRRSV dsRNA. (E) Cells were infected with PRRSV BJ-4 at an MOI of 10 for 24 h. The efficiency of viral assembly in the supernatants was determined by comparing the infectious titers (TCID_50_ per milliliter) with the total PRRSV genome equivalents (GE). *, *P < *0.05 (by an unpaired two-tailed *t* test). (F) Cells were infected with PRRSV BJ-4 at an MOI of 10 for 24 h. The efficiency of virus secretion was determined as the ratio of intra- and extracellular infectivity relative to the total infectivity. NS, not significant (by an unpaired two-tailed *t* test).

We next investigated the impact of NDRG1 knockdown on the stages of the PRRSV life cycle. Cells expressing sh-control or sh-NDRG1 were incubated on ice with a high multiplicity of infection (MOI) of PRRSV for 1 h, and viral binding was assayed by RT-qPCR. We saw no significant differences between viral binding to sh-control-expressing cells and cells depleted of NDRG1; the levels of binding were virtually indistinguishable (Fig. 5A). To determine whether NDRG1 deficiency affected viral internalization, cells were incubated with PRRSV on ice and then shifted to 37°C for 2 h. Viral internalization was then assayed by RT-qPCR. As shown in Fig. 5B, no significant change in viral entry into sh-NDRG1 cells was observed after a temperature shift to 37°C for 2 h. To examine PRRSV replication, cells were infected with PRRSV for 6, 12, or 24 h and then fixed and stained for double-stranded RNA (dsRNA), as a marker of viral replication (Fig. 5C). NDRG1 knockdown significantly increased PRRSV dsRNA levels as early as 6 h in the sh-NDRG1-transfected cells (10 random fields of view; *n* > 100 cells each). The numbers of cells that stained positive for dsRNA were also larger than in the control cells at 12 and 24 h (Fig. 5D). To further assess the role of NDRG1 in PRRSV particle production, the specific infectivity in the cell supernatants was determined by comparing the infectious titers with the viral RNA levels. There was a significant increase in PRRSV particle production when the cells expressed lentivirus-derived sh-NDRG1 (lenti-sh-NDRG1) (Fig. 5E). However, the relative amounts of intra- and extracellular infectivity relative to the total infectivity did not change (Fig. 5F), suggesting that silencing NDRG1 expression affected the assembly but not the release of the PRRSV particles. Therefore, we conclude that NDRG1 depletion predominantly affects PRRSV replication during the PRRSV life cycle, although a secondary effect on particle assembly cannot be excluded.

### NDRG1 knockdown reduces cellular LDs.

**FIG 6:**
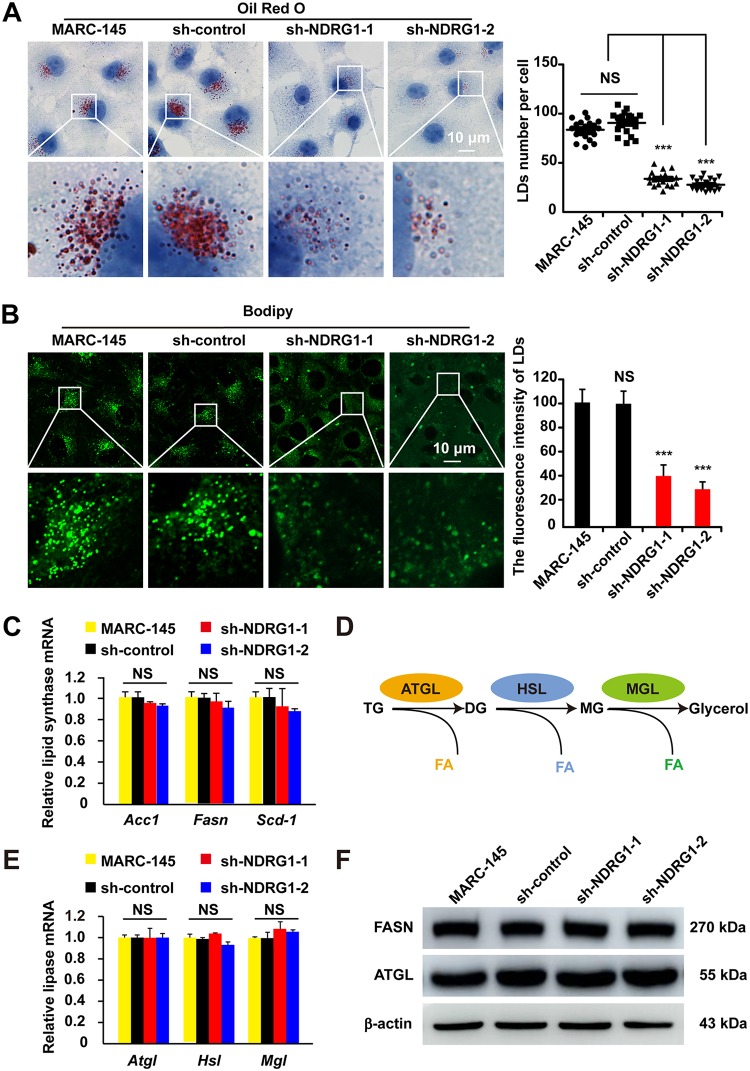
NDRG1 knockdown reduces intracellular LD numbers. (A) Cells were fixed and stained with oil red O. Nuclei were counterstained with hematoxylin. The boxed region in the top image is enlarged at the bottom. Each image is representative of results from three independent experiments. A plot of the LD numbers per cell in the oil red O images is shown. At least 80 cells were counted. NS, not significant; ***, *P < *0.0001 (by one-way ANOVA). (B) LDs in cells were stained with BODIPY 493/503. The boxed region in the top image is enlarged at the bottom. Each image is representative of results from three independent experiments. ImageJ analysis was used for the semiquantification of the fluorescence intensity. NS, not significant; ***, *P < *0.0001 (by one-way ANOVA). (C) *ACC1*, *FASN*, and *SCD* mRNA levels were examined by RT-qPCR in MARC-145 cells and shRNA-expressing cells. Values were normalized to β-actin (*ACTB*) mRNA levels. NS, not significant (by one-way ANOVA). (D) Schematic diagram of the coordinated breakdown of TG. (E) *ATGL*, *HSL*, and *MGL* mRNA levels were examined by RT-qPCR in MARC-145 cells and shRNA-expressing cells. Values were normalized to β-actin (*ACTB*) mRNA levels. NS, not significant (by one-way ANOVA). (F) Immunoblot analyses of FASN and ATGL expression in MARC-145 cells and shRNA-expressing cells. β-Actin was used as the loading control.

NDRG1 is reported to restrict HCV propagation by regulating LD biogenesis (29). Therefore, we examined whether LDs were modified in sh-NDRG1-expressing MARC-145 cells using oil red O and BODIPY 493/503 staining. By microscopy, we found that the LD numbers decreased by ∼70% compared with those in the control cells (*P < *0.0001) (Fig. 6A). The intensity of LD staining in the cytoplasm of the sh-NDRG1-expressing cells was also substantially reduced (Fig. 6B). We hypothesized that this was caused by reduced TG synthesis or enhanced lipolysis. To test this, we examined the expression of key lipogenic genes, including *ACC1*, *FASN*, and *SCD-1*, in the NDRG1 knockdown cells. However, the mRNA levels of *ACC1*, *FASN*, and *SCD-1*, which are involved in fatty acid biosynthesis, were not altered (Fig. 6C). Therefore, we inferred that NDRG1 deficiency reduced the number of LDs through increased lipolysis. The coordinated breakdown of TGs (lipolysis) is summarized in Fig. 6D. The rate-limiting step is the cleavage of the first ester bond in TGs by adipose triglyceride lipase (ATGL), producing diacylglycerol (DG) and releasing one FA. Hormone-sensitive lipase (HSL) hydrolyzes DG to monoacylglycerol (MG), and monoglyceride lipase (MGL) then hydrolyzes MG to glycerol and FA. However, the mRNA levels of the lipolysis genes *ATGL*, *HSL*, and *MGL* showed no obvious changes in MARC-145 cells after NDRG1 knockdown (Fig. 6E). We also assessed the fatty acid synthase (FASN) and ATGL protein levels after NDRG1 depletion, and our results showed that sh-NDRG1 did not significantly affect the protein levels of FASN or ATGL (Fig. 6F). In summary, NDRG1 plays an important role in regulating lipid storage, and its deficiency reduces lipid storage, but the expression levels of key lipogenic and lipolytic genes remain unchanged. This raises the question, By what mechanisms are these LDs so dramatically decreased?

### NDRG1 depletion promotes autophagy and increases cellular FFA levels.

**FIG 7:**
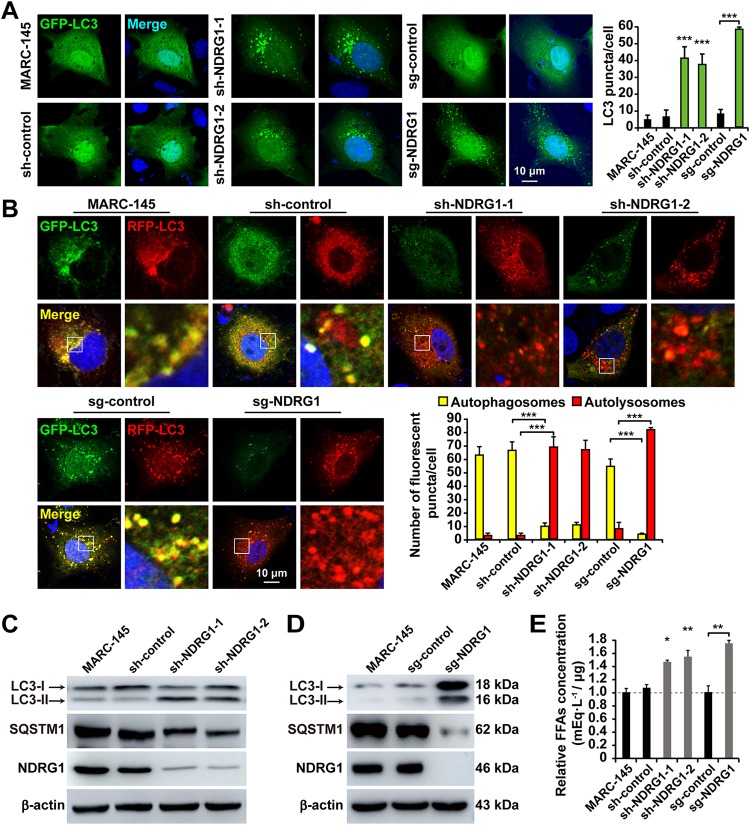
NDRG1 deficiency induces autophagy and increases the FFA content. (A) Cells were transfected with a plasmid encoding GFP-LC3 for 24 h and then fixed and stained with DAPI (blue). The number of GFP-LC3 puncta per cell was counted in 30 cells per group. ***, *P < *0.0001 (by one-way ANOVA). (B) Cells were transfected with a plasmid encoding RFP-GFP-LC3 for 24 h and then fixed and stained with DAPI. The boxed region in the image is enlarged below. The numbers of autophagosomes (RFP- and GFP-positive puncta) and autolysosomes (only RFP-positive puncta) per cell were counted in 30 cells per group. ***, *P < *0.0001 (by one-way ANOVA). (C) Immunoblot analysis of LC3 and SQSTM1 expression in MARC-145 cells and shRNA-expressing cells. β-Actin was used as the loading control. (D) Immunoblot analysis of LC3 and SQSTM1 expression in MARC-145 cells and NDRG1^−/−^ cells. β-Actin was used as the loading control. (E) Cellular lipids were extracted from cells, and the amounts of FFAs were quantified. Values were normalized to the total cellular protein content. *, *P < *0.05; **, *P < *0.01 (by one-way ANOVA).

Autophagy is a cellular recycling mechanism that provides cells with a source of energy during periods of nutrient insufficiency (51). Recent studies have shown a role of autophagy in LD breakdown, thus providing energy more efficiently from the hydrolyzed FFAs (52–54). The direct contribution of autophagy-mediated lipid mobilization is termed “lipophagy.” Because lipophagy is involved in LD catabolism (55), we investigated the links between the reduced LD numbers and autophagy or lipophagy in sh-NDRG1 cells. Microtubule-associated protein light chain 3-II (LC3-II), the phosphatidylethanolamine-conjugated form of LC3, is present in autophagosomes and is therefore commonly used as a marker of autophagosome formation. Cytoplasmic LC3 puncta are characteristic of autophagosomal membrane formation. Therefore, we examined whether NDRG1 deficiency induced LC3 puncta by ectopically expressing GFP-LC3 from a plasmid in cells. As shown in Fig. 7A, GFP-LC3 showed a diffuse distribution pattern in MARC-145 and control cells, whereas it was arranged as punctuate cytoplasmic dots in the sh-NDRG1- and sg-NDRG1-expressing cells. To measure autophagic flux, we used the tandem GFP-red fluorescent protein (RFP)-LC3 sensor (56), which contains acid-labile GFP and acid-resistant RFP, to distinguish autophagosomal and autolysosomal localizations. In this assay, GFP- and RFP-tagged LC3 detected autophagosomes, whereas RFP detected only autolysosomes because GFP is denatured in the acidic environment of the autolysosome. Thus, in the superimposed fluorescence images, yellow dots represent autophagosomes, and red dots represent autolysosomes. We observed an increased RFP signal relative to the GFP signal in response to NDRG1 deficiency, indicating enhanced autophagic flux after the depletion of NDRG1 (Fig. 7B). NDRG1 deficiency increased LC3-II formation but decreased SQSTM1 relative to its levels in MARC-145 and sh/sg-control cells (Fig. 7C and D), thus suggesting that the autophagy pathway was activated in the NDRG1-deficient cells. Because autophagy has been implicated in the lipolysis of LDs, we investigated whether NDRG1 deficiency-induced autophagy promoted the production of FFAs. Notably, the amount of total cellular FFAs was significantly larger in the NDRG1 knockdown and knockout cells than in the control cells (Fig. 7E). Collectively, these findings support the proposition that NDRG1 deficiency promotes autophagy and consequently increases the yield of hydrolyzed FFAs.

### Autophagy is required for LD depletion and increased PRRSV replication.

**FIG 8:**
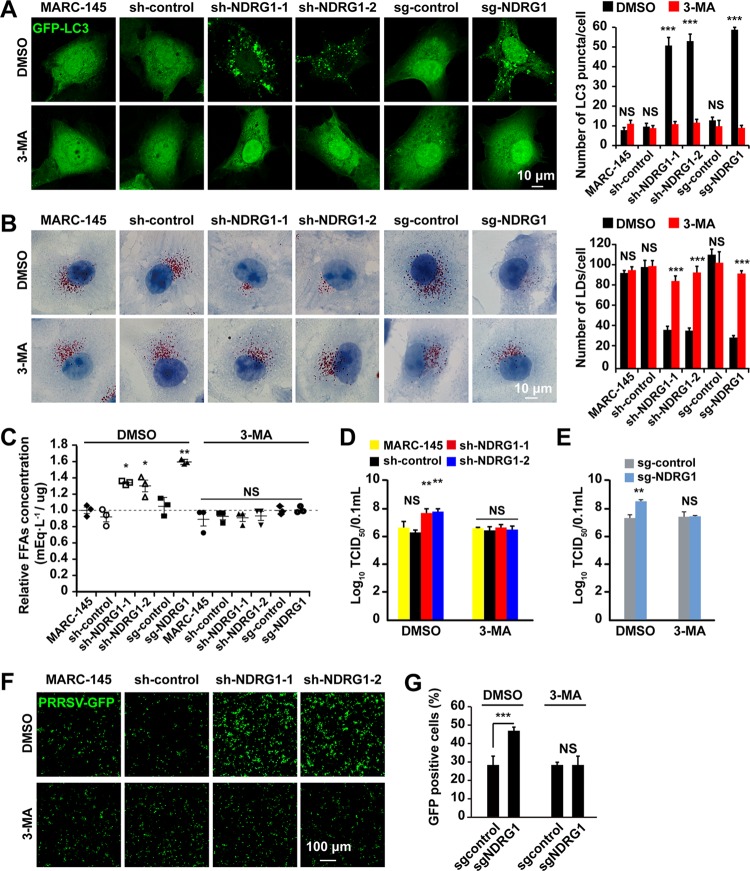
Inhibition of autophagy abrogates the promotion of PRRSV replication. (A) Cells transfected with a plasmid encoding GFP-LC3 were treated with DMSO or 3-MA for 24 h. The number of GFP-LC3 puncta per cell was counted in 30 cells per group. NS, not significant; ***, *P < *0.0001 (by one-way ANOVA). (B) Cells were treated with DMSO or 3-MA for 24 h and then fixed and stained with oil red O. Nuclei were counterstained with hematoxylin. The average number of LDs per cell was counted in 30 cells per group. NS, not significant; ***, *P < *0.0001 (by one-way ANOVA). (C) Cells were treated with DMSO or 3-MA for 24 h. Cellular lipids were extracted, and the amounts of FFAs were quantified. Values were normalized to the total cellular protein content. NS, not significant; *, *P < *0.05 (by one-way ANOVA). (D) MARC-145 and shRNA-expressing cells were infected with PRRSV BJ-4 (MOI = 10) for 1 h. The virus was removed, and 3-MA was applied at 48 h postinfection. Virus was harvested with three freeze-thaw cycles, and the viral titer was determined by a TCID_50_ assay. NS, not significant; **, *P < *0.01 (by one-way ANOVA). (E) sg-control and NDRG1^−/−^ cells were infected with PRRSV BJ-4 (MOI = 10) for 1 h. The virus was removed, and 3-MA was applied at 48 h postinfection. Virus was harvested with three freeze-thaw cycles, and the viral titer was determined by a TCID_50_ assay. NS, not significant; **, *P < *0.01 (by one-way ANOVA). (F) MARC-145 and shRNA-expressing cells were infected with PRRSV-GFP (MOI = 10) for 1 h. The virus was removed, and 3-MA was applied at 48 h postinfection. Fluorescence was detected with a fluorescence microscope. The bottom panel shows bright-field images of the corresponding cells. (G) sg-control and NDRG1^−/−^ cells were infected with PRRSV-GFP (MOI = 10) for 48 h, and the fluorescence-positive cells were measured by flow cytometry. ***, *P < *0.0001 (by one-way ANOVA).

We next tested whether autophagy is required for the changes in LD numbers and the promotion of PRRSV replication in sh/sg-NDRG1-expressing cells. Cells were transfected with the GFP-LC3-expressing plasmid, as described above, and treated with 3-methyladenine (3-MA), a well-characterized inhibitor of autophagy. The effects of 3-MA on autophagy were detected as the formation of cytoplasmic LC3 puncta by fluorescence microscopy. In the vehicle-treated NDRG1 knockdown and knockout cells, the number of LC3 puncta was significantly elevated, whereas in the cells treated with 3-MA, the number of puncta was reduced (Fig. 8A). These results indicate that the autophagy induced by NDRG1 deficiency was inhibited by pharmacological treatment. To further examine NDRG1 function, we measured the amount of LDs in the presence of 3-MA. In dimethyl sulfoxide (DMSO)-treated cells, the LD number was again lower in sh/sg-NDRG1-expressing cells than in MARC-145 and sh/sg-control cells. The inhibition of autophagy significantly increased the areas of LDs in the NDRG1-deficient cells (Fig. 8B). To examine the potential effect of autophagy on the yield of cellular FFAs, we treated cells with 3-MA. FFAs were depleted in the sh/sg-NDRG1-expressing cells after autophagy was inhibited by 3-MA treatment (Fig. 8C). Next, PRRSV replication was analyzed in the presence of 3-MA. MARC-145 and sh/sg-NDRG1-expressing cells were pretreated with DMSO or 3-MA for 24 h before they were infected with PRRSV BJ-4. The titer assay results showed that 3-MA treatment significantly abrogated the increased viral replication in NDRG1-deficient cells (Fig. 8D and E). Similarly, 3-MA treatment also significantly decreased the proliferation of PRRSV-GFP in NDRG1-deficient cells (Fig. 8F and G). Collectively, these findings confirm that autophagy plays a critical role in the lipolysis of LDs and in PRRSV replication.

## DISCUSSION

Viruses hijack host cellular components and metabolic networks to improve their survival. The host factors that are responsive to viral infection and regulate such metabolic changes are largely unknown. However, knowledge of these factors is essential for understanding viral infection and can offer potential targets for antiviral therapies. NDRG1 has been implicated in many cellular functions, including stress responses (17), cell growth (19), cell differentiation (18), vesicular trafficking (21), apoptosis (22), and autophagy (28). It is also reported to restrict HCV propagation (29) but to facilitate IAV replication (30). Therefore, the molecular mechanisms underlying the involvement of NDRG1 in viral replication vary markedly. In this study, we identified a function of NDRG1 in PRRSV infection insofar as it regulates lipid metabolism by regulating autophagy.

We have shown that *NDRG1* mRNA is ubiquitously expressed in different pig tissues but most strongly in white adipose tissue (Fig. 1), suggesting that NDRG1 may be involved in lipid metabolism. Previous research has shown that NDRG1 plays an important role in cholesterol metabolism (57). Low-density lipoprotein (LDL) particles are transported to lysosomes for hydrolysis into free cholesterol, which is then released by Niemann-Pick type C2 (NPC2) and NPC1. Interestingly, a genomewide expression analysis of fibroblasts derived from a patient with Niemann-Pick type C disease showed that *NDRG1* mRNA levels were elevated (58). Moreover, RNA interference (RNAi) screening focusing on cellular lipid biogenesis suggested that NDRG1 affects cholesterol homeostasis (59). Furthermore, silencing of NDRG1 in epithelial cells reduced the uptake of LDL, suggesting that NDRG1 functions in LDL receptor trafficking by regulating endosomal recycling and degradation (57). However, the mechanisms by which NDRG1 regulates cholesterol and lipid homeostasis remain unknown.

At the subcellular level, our fluorescence analysis indicated that NDRG1 does not colocalize with peroxisomes, lysosomes, actin, or mitochondria but mainly colocalizes with the ER (data not shown). This observation is consistent with the finding that NDRG1 is a predominantly cytosolic protein, expressed ubiquitously in normal and neoplastic tissues (60). The ER is an organelle involved in protein synthesis and modification, so it also plays an essential role in viral replication and maturation. In the course of virus proliferation, large amounts of viral proteins are synthesized, and unfolded or misfolded proteins cause ER stress. During hepatitis B virus (HBV) and HCV infection, the virus triggers autophagosome formation by inducing ER stress (61, 62). It has been demonstrated that NDRG1 overexpression inhibits the initiation of basal autophagy and the ER-stress-mediated autophagy pathway in cancer cells (28). This implies a close relationship between the ER localization of NDRG1 and its function in suppressing ER-stress-mediated autophagy. Thus, the subcellular localization of NDRG1 on the ER may help us understand its function.

In this study, we have shown that PRRSV infection reduces NDRG1 mRNA and protein expression (Fig. 2). The NDRG1 deficiency increased PRRSV replication (Fig. 3), whereas the overexpression of NDRG1 reduced viral RNA and protein production (Fig. 4), suggesting that NDRG1 acts as a limiting factor for PRRSV infection. Interestingly, it has been reported that NDRG1 is associated with viral proliferation, but it has different functions in HCV and IAV replication. HCV is an enveloped, single-stranded, positive-sense RNA virus, and NDRG1 restricts HCV assembly by limiting LD formation. HCV counteracts this intrinsic antiviral mechanism by downregulating NDRG1 via a MYC-dependent mechanism (29). Conversely, the IAVs are enveloped viruses with a single-stranded, negative-sense RNA genome, and recent evidence suggests that NDRG1 plays a positive role in avian influenza (highly pathogenic avian influenza [HPAI]) A/H5N1 virus replication by suppressing the canonical NF-κB signaling pathway (30). PRRSV is an enveloped, single-stranded, positive-sense RNA virus, and we have shown that NDRG1 plays a negative role in PRRSV replication. The distinct functions of NDRG1 in the positive- and negative-stranded RNA viruses prompt the suggestion that NDRG1 inhibits the replication of positive-stranded viruses and promotes the replication of negative-stranded viruses. If so, what role does NDRG1 play in the pathogenesis of DNA viruses? These questions must be addressed in future research.

Various viruses hijack host LDs to complete their own life cycles. HCV (63), rotaviruses (64), and DENV (65) are known to use LDs as platforms for viral assembly. Because they contain hydrophobic neutral lipids at their cores, LDs are an efficient storage site for energy (34). DENV replication activates the autophagic pathway to mobilize the FFAs from LDs. The FFAs released from LDs are consumed by oxidation in mitochondria to generate ATP, which is a tremendous reservoir that supplies energy for viral replication (37). In previous studies, it has been reported that PRRSV enters cells via a receptor-mediated mechanism. However, R. Guo et al. found that PRRSV can also use intercellular nanotubes for transporting infectious viral materials (viral RNA, certain replicases, and certain structural proteins) into the cytosol of a neighboring cell (66). Our findings show that NDRG1 depletion enhances PRRSV RNA replication and progeny virus assembly (Fig. 5). Therefore, these increased RNAs and progeny viruses by reduced NDRG1 expression may increase PRRSV infection by enhancing cell-to-cell spread.

It has also been shown that LD numbers were significantly increased in NDRG1 knockdown Huh7 (hepatocarcinoma) cells (29). Therefore, we assessed whether the loss of NDRG1 affects the fatty acid metabolism of cultured monkey kidney (MARC-145) cells. As shown in Fig. 6, LD numbers and their fluorescence intensity were substantially reduced in NDRG1-deficient cells. To confirm our results, six different shRNAs were designed to target NDRG1, and all produced the same phenotype of reduced LD numbers (the data for the other four shRNAs are not shown). It is possible that these conflicting results are attributable to the different cell types examined in each study. Huh7 cells are liver cancer cells and MARC-145 cells are monkey kidney cells, and the differences between cancer cells and normal cells are vast. We also demonstrated here that the function of NDRG1 in lipid metabolism does not seem to be mediated by the expression of key lipogenic or lipolytic genes (Fig. 6C to F). Therefore, it is important to understand the functions of NDRG1 in different cell types and the roles that it plays in lipid metabolism and viral replication.

An association between autophagy and lipolysis has been reported (67, 68), and the loss of ATG7 causes LDs to accumulate in hepatocytes (69). In this study, we identified a new function of NDRG1 in regulating autophagy and lipid metabolism. Fluorescence and immunoblot analyses indicated that NDRG1 deficiency led to the activation of autophagic flux and increased the intracellular FFA content (Fig. 7). The inhibition of autophagy by 3-MA restored the spatial area of LDs, reduced the FFA content, and inhibited the promotion of PRRSV replication (Fig. 8). Therefore, autophagy not only regulates the cellular LD content but also supplies the virus with FFAs to complete its intracellular replication and assembly cycles. The FFAs released from the degradation of TGs undergo mitochondrial β-oxidation to produce energy. This process is frequently manipulated by flaviviruses to promote their replication (37). Our unpublished data also indicate that etomoxir, a drug that inhibits β-oxidation by preventing the transport of FFAs into the mitochondria, inhibited PRRSV-GFP replication in a dose-dependent manner. Therefore, NDRG1 is probably a crucial host factor in the nexus of PRRSV infection and the autophagy-mediated processing of cellular LDs.

**FIG 9:**
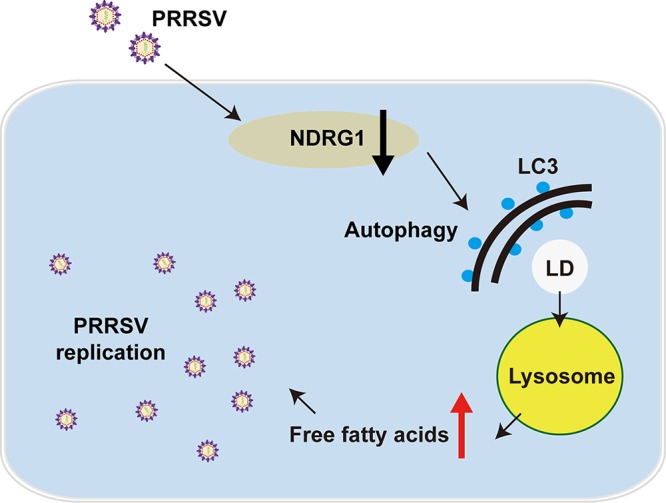
NDRG1 deficiency induces autophagy, which alters cellular lipid metabolism, promoting PRRSV replication. PRRSV infection reduced the expression of NDRG1, and the loss of NDRG1 induced autophagy. Autophagy provides FFAs via LD degradation, which increases the virus yield.

Overall, our study demonstrates a previously unreported relationship between PRRSV, NDRG1, and lipophagy in the context of viral infection. Our model indicates that PRRSV infection reduces NDRG1 expression and that the loss of NDRG1 induces autophagic flux. Lipophagy degrades LDs to produce the FFAs required to increase the viral yield (Fig. 9). NDRG1 appears to restrict PRRSV infection by suppressing the LD degradation that is necessary for PRRSV replication. PRRSV has evolved a mechanism to counteract this restriction by downregulating NDRG1 expression, resulting in enhanced lipophagy. Thus, NDRG1-deficient cells could be used in vaccine production instead of traditional cells because they allow a higher viral titer to be achieved. Further work is required to determine the relationship between NDRG1 and the pathogenesis of PRRSV, to promote the development of novel antiviral strategies against PRRSV infection.

## MATERIALS AND METHODS

### Materials.

We obtained SYBR premix Ex *Taq* (catalogue number RR420A) and TRIzol reagent (catalogue number D9108B) from TaKaRa Bio Inc. (Otsu, Shiga, Japan); the TIANamp virus RNA/DNA kit (catalogue number DP315-R) was obtained from Tiangen (Beijing, China); anti-LC3 antibody (catalogue number 4599), anti-SQSTOM/P62 antibody (catalogue number 5114), anti-NDRG1 antibody (catalogue number 9485), and anti-ATGL antibody (catalogue number 2138S) were obtained from Cell Signaling Technology (Danvers, MA, USA); anti-PRRSV nucleocapsid (N) antibody SDOW17 was obtained from Rural Technologies (WA, USA); mouse monoclonal anti-dsRNA antibody was obtained from English & Scientific Consulting (Szirak, Hungary); the cell counting kit (catalogue number ZP328) was obtained from Zoman Bio (Beijing, China); oil red O was obtained from Sigma-Aldrich (St. Louis, MO, USA); BODIPY 493/503 (catalogue number D3922), anti-mouse IgG antibody labeled with Alexa Fluor 555 (catalogue number A21424) and Alexa Fluor 488 (catalogue number A21429), and anti-rabbit IgG antibody labeled with Alexa Fluor 488 (catalogue number A11034) were obtained from Thermo Fisher Scientific (Waltham, MA, USA); 3-methyladenine (catalogue number 5142-23-4) was obtained from Med Chem Express (Monmouth Junction, NJ, USA); the LabAssay nonesterified fatty acids (NEFA) (catalogue number 294-63601) assay kit for free fatty acids was obtained from Wako Bioproducts (Richmond, VA); and horseradish peroxidase (HRP)-conjugated donkey anti-mouse IgG (catalogue number 715-035-150) and anti-rabbit IgG (catalogue number 711-035-152) antibodies were obtained from Jackson ImmunoResearch Laboratories (West Grove, PA, USA). The antibodies described above were used at dilutions of 1:500 for immunofluorescence staining and 1:1,000 for immunoblotting.

### Plasmids and transient transfection.

The coding sequence of porcine *NDRG1* was amplified from porcine white adipose cDNA and cloned into the p3×FLAG-CMV-10 (Sigma, St. Louis, MO) or pmCherry-C1 plasmid. The primers are listed in Table 1. The plasmids pEGFP-LC3 (catalogue number 24920) and pMRX-IP-GFP-LC3-RFP (catalogue number 84573) were purchased from Addgene (Cambridge, MA, USA). All plasmids were transfected with Lipofectamine 3000 (Invitrogen, Grand Island, NY), according to the manufacturer’s instructions.

**TABLE 1:** Primers used for gene cloning and RT-qPCR

Primer	Sequence (5′–3′)	Product size (bp)
Flag-NDRG1	GGGGTACCAATGTCTCGGGAGCTGCAGGA	1,155
	CGGGATCCTTAGCAGGACACCTCCGCAGA	

Q-Sus NDRG1	ACACCTACCGACAGCACATC	177
	CGTCCACAGCAGGAGAATTG	

Q-sus β-actin	CTGAACCCCAAAGCCAACCGT	317
	TTCTCCTTGATGTCCCGCACG	

Q-PRRSV ORF7	AGATCATCGCCCAACAAAAC	144
	GACACAATTGCCGCTCACTA	

Q-Chlorocebus β-actin	CGTGGACATCCGTAAAGAC	182
	GGAAGGTGGACAGCGAGGC	

Q-Chlorocebus ACC	TAGTCTGCCACGGATCCAGA	179
	GGGAGGGATCTCTGAGGGTT	

Q-Chlorocebus FASN	CACATCGTTCGAGCAGCATG	133
	AATTTCCAGGAAGCGACCGT	

Q-Chlorocebus SCD	AGGGCCCCAATGTATGTGTG	196
	AAAGATGTAAGGCACCCGGG	

Q-Chlorocebus ATGL	GAGATGTGCAAGCAGGGCTA	120
	ACTCTCCATGGCCTCATCCT	

Q-Chlorocebus HSLL	CCTCCGGGAGTATGTTACGC	113
	ACACCAGCCCAATGGAGATG	

Q-Chlorocebus MGL	GTCTTCCTTCTGGGCCACTC	159
	GTTGAGCACTTTCGCAGCAA	

Q-Chlorocebus NDRG1	ACGCACGAACACACAAACGG	135
	TCACCTCAGCGAGGTCTACG	

### Cells, viruses, and tissues.

MARC-145, HEK293T, and 3D4/21-CD163 (generated through lentivirus-mediated overexpression of porcine CD163) cells were grown in monolayers at 37°C under 5% CO_2_ and maintained in Dulbecco’s modified Eagle’s medium (DMEM; Gibco, Grand Island, NY) containing 100 U/ml penicillin and 100 μg/ml streptomycin sulfate, which was supplemented with 10% heat-inactivated fetal bovine serum (FBS; PAN, Aidenbach, Germany). Porcine alveolar macrophages (PAMs) were obtained from 6-week-old PRRSV-negative piglets through a lung lavage method, as described previously (70), and cultured in RPMI 1640 supplemented with 10% FBS and penicillin-streptomycin.

The recombinant PRRSV-GFP strain (71) was kindly donated by En-Min Zhou from Northwest A&F University (Yangling, Shaanxi, China). The PRRSV BJ-4 strain was used as described previously (72).

Male 30-day-old Sanyuan pigs (three-way crossbreeds [Landrace × Largewhite × Duroc]) seronegative for porcine circovirus type 2, PRRSV, and mycoplasma (73) were sacrificed to obtain the experimental tissues, including the liver, heart, spleen, kidney, lymph nodes, skin, white adipose tissue, muscle, duodenum, jejunum, ileum, and cecum. All tissues were immediately snap-frozen in liquid nitrogen, stored at −80°C, and used as described previously (74). All animal procedures were authorized and supervised according to rules that were approved by the State Council of the People's Republic of China for experimental animal care and use.

### Immunoblot analysis.

Whole-cell lysates were prepared in radioimmunoprecipitation assay (RIPA) buffer (50 mM Tris-HCl [pH 8.0], 150 mM NaCl, 1% Triton X-100, 1% sodium deoxycholate, 0.1% SDS, 2 mM MgCl_2_) supplemented with protease inhibitors (Roche). Protein samples were separated by SDS-PAGE and then transferred to cellulose nitrate membranes. After incubation in 5% nonfat milk for 30 min, the membrane was incubated with the primary antibody overnight at 4°C and then with the horseradish peroxidase-conjugated secondary antibody for 1 h at room temperature. The target proteins were detected with the Luminata Crescendo immunoblotting HRP substrate (Millipore, Billerica, MA). Each experiment was repeated at least three times using separate batches of cells.

### Immunofluorescence.

Cells grown on coverslips were fixed with 4% paraformaldehyde (PFA) for 30 min, permeabilized in 0.1% Triton X-100, and then incubated with phosphate-buffered saline (PBS) containing 10% FBS (10% FBS–PBS) with the primary antibody (1:500) for 1 h at room temperature. After the cells were washed three times with PBS, they were labeled with 10% FBS–PBS containing the fluorescent secondary antibody (1:500) for 1 h. Images were acquired using a Zeiss LSM710 confocal microscope. Digital images were taken with ZEN 2012 software and processed with ImageJ (National Institutes of Health). The images were quantified with ImageJ and a set of defined intensity thresholds that were applied to all images.

### Reverse transcription-quantitative PCR.

Total RNA was extracted from cells or 1 ml of the cell culture supernatant with TRIzol reagent or the TIANamp virus RNA/DNA kit, respectively. The RNA was then reverse transcribed into cDNA with an oligo(dT) or random hexamer primer. qPCR was performed with the Eppendorf Mastercycler ep realplex real-time PCR system, according to the manufacturer’s protocol. All reactions were performed in triplicate, and the relative amounts of mRNAs were calculated with the comparative threshold cycle (*C_T_*) method. The results obtained were representative of data from three independent experiments. Serial 10-fold dilutions of a plasmid containing PRRSV open reading frame 7 (ORF7), which encodes the viral N protein, were used to construct a standard curve. The total number of genomic equivalents was determined by comparison with the standard curve.

### Production of cells stably expressing NDRG1 shRNA.

Short hairpin RNA (shRNA) sequences targeting Chlorocebus sabaeus NDRG1 were selected with BLOCK-iT RNAi designer (Life Technologies, Carlsbad, CA) (sh1 [GCAGGACATCGAGACTTTACA] and sh2 [CCTACATCCTAACTCGATTT]). Lentiviral particles were then produced. Briefly, 4 × 10^6^ human HEK293T cells were plated on 10-cm dishes for 24 h before they were transfected with 1 μg of the shRNA-containing pLKO.1 vector, 0.25 μg of pMD2.G (envelope-expressing plasmid), and 0.75 μg of psPAX2 (packaging-expressing plasmid). Transfection was performed with 3 volumes of Lipofectamine (microliters) to 1 volume of DNA (micrograms). The medium was replaced after 6 h. Viral particles were collected 48 h after transfection and used to infect MARC-145 cells. The infected cells were selected in medium containing 10 μg/ml puromycin for 1 week.

### CRISPR/Cas9-mediated knockout of NDRG1 in MARC-145 cells.

To disrupt the NDRG1 gene, we designed a CRISPR/Cas9 single guide RNA system to target exon 4 of NDRG1, by using the optimized design software at http://crispr.mit.edu. The 20-nucleotide (nt) guide sequence (T1, 5′-GCGCTGTGTGGGACTCCCAA-3′; T2, 5′-GCGTTCACGTCACGCTGTGT-3′ or 5′-GCCGTTCACGTCACGCTGTG-3′) was cloned into pLentiCRISPRv2 containing a Cas9 expression cassette. Lentivirus particles harboring Cas9 alone (sg-control) or Cas9/NDRG1 sgRNA (sg-NDRG1) were transduced into MARC-145 cells. After selection with 10 μg/ml puromycin, sg-control was pooled, and single clones stably expressing sg-NDRG1 were isolated. To identify the status of genome editing, we performed PCR amplification of the genomic DNA isolated from different clonal cell lines by using primers specific for the target sequence and then performed DNA sequencing. The efficiency of NDRG1 knockout was determined by immunoblotting with NDRG1 antibody.

### Cell proliferation assay.

Cell viability was evaluated with the cell counting kit (CCK) assay, according to the manufacturer’s instructions. In brief, MARC-145 cells and sh-NDRG1-expressing cells were seeded into 96-well plates with 0.8 × 10^4^ cells/well for the indicated time periods. CCK (10 μl) was then added to each well, and the cells were incubated for 3 h at 37°C. The absorbance at 450 nm was detected with a microplate reader (Awareness Technology Inc.).

### Viral titration and infectivity.

A 50% tissue culture infective dose (TCID_50_) assay was performed to assess viral titration and infectivity. On day 0, MARC-145 cells were seeded in a 96-well plate at a density of 1 × 10^4^ cells per well. On day 1, the cells were inoculated with serially 10-fold diluted viruses at 37°C for 1 h. The excess virus inoculum was removed by washing the cells with PBS. Maintenance medium (2% FBS–DMEM; 200 μl) was added to each well, and the cells were cultured for a further 3 to 5 days. Cells showing the expected cytopathic effect were counted daily, and the TCID_50_ value was calculated with the Reed-Muench method (75). Each assay was performed in triplicate.

**(i) Intracellular infectivity.** Cells were washed three times with PBS, scraped, and pelleted by centrifugation at 1,000 × *g* for 5 min. The cell pellets were subjected to three cycles of freeze-thawing with liquid nitrogen and a thermal block set to 37°C. The cell lysates were centrifuged at 10,000 × *g* for 10 min at 4°C to remove the cell debris. The titers of infectious virus were expressed as TCID_50_ per milliliter after a limiting-dilution assay.

**(ii) Extracellular infectivity.** To determine the extracellular infectivity of the virus, the supernatants were harvested, filtered through a 0.45-μm-pore-size filter, and stored at 4°C. Their infectivity was determined in parallel with a limiting-dilution assay, as described above.

### LD staining.

Cells were washed with PBS and fixed in 4% PFA for 30 min. After the cells were washed again with PBS, they were stained with oil red O (saturated oil red O solution in isopropanol-water at a 3:2 dilution) for 15 min. The cells were then washed with 70% alcohol for 5 s to remove any background stain, rinsed in double-distilled Millipore water, counterstained with Harris hematoxylin (10 s), washed, mounted, and observed under a light microscope. The LD number was determined with the ImageJ “analyze particles” function (areas of particles of <0.01 mm^2^ were excluded).

The cells were washed with PBS and fixed in 4% PFA for 30 min. After the cells were washed again with PBS, they were incubated with 2 μg/ml BODIPY 493/503 (493-nm excitation/503-nm emission) for 30 min. Digital images were obtained with a Zeiss LSM710 confocal microscope. Fluorescence intensity was determined with ImageJ software.

### Flow cytometry.

MARC-145 and *NDRG1* shRNA-expressing cells were infected with PRRSV-green fluorescent protein (PRRSV-GFP) at a multiplicity of infection (MOI) of 10. Forty-eight hours after viral infection, the cells were washed with PBS and digested with a trypsin-EDTA solution. The cells were then collected by centrifugation, washed twice with ice-cold PBS, and resuspended in 0.4 ml of fresh PBS. A flow cytometric analysis was performed to monitor the GFP-positive cells using a Beckman CytoFLEX flow cytometer. All data were analyzed with CytExpert software.

### Determination of intracellular FFAs.

FFAs were measured with the LabAssay NEFA kit (Wako). The intracellular FFAs assay was performed according to the manufacturer’s instructions. In brief, after infection or treatment, the cell lysates were extracted with a syringe needle in 250 μl of RIPA buffer and centrifuged at 12,000 × *g* for 5 min at 4°C. The total lipids in 200 μl of the lysate were extracted by the addition of 100 μl of a chloroform-methanol (2:1, vol/vol) mixture. The extract was evaporated to dryness and dissolved in 50 μl of TRB (100 mM KH_2_PO_4_, 100 mM K_2_HPO_4_, 5 mM sodium cholate, 50 mM NaCl, 0.1% Triton X-100 [pH 7.4]) for the FFA assay. The values were normalized to the total cellular protein content, which was determined with a Bio-Rad protein assay kit (catalogue number 5000001).

### Statistical analysis.

Data were obtained from at least three independent experiments for the quantitative analyses and are expressed as means ± standard errors of the means. All statistical analyses were performed with a *t* test or one-way analysis of variance (ANOVA). Significant differences were accepted at *P* values of *<*0.05, *<*0.01, and *<*0.0001 versus the corresponding controls.

## References

[1] Lunney JK, Benfield DA, Rowland RR 2010 Porcine reproductive and respiratory syndrome virus: an update on an emerging and re-emerging viral disease of swine. Virus Res 154:1–6. doi:10.1016/j.virusres.2010.10.009. 20951175 PMC7172856

[2] Rossow KD 1998 Porcine reproductive and respiratory syndrome. Vet Pathol 35:1–20. doi:10.1177/030098589803500101. 9545131

[3] Snijder EJ, Kikkert M, Fang Y 2013 Arterivirus molecular biology and pathogenesis. J Gen Virol 94:2141–2163. doi:10.1099/vir.0.056341-0. 23939974

[4] Tian K, Yu X, Zhao T, Feng Y, Cao Z, Wang C, Hu Y, Chen X, Hu D, Tian X, Liu D, Zhang S, Deng X, Ding Y, Yang L, Zhang Y, Xiao H, Qiao M, Wang B, Hou L, Wang X, Yang X, Kang L, Sun M, Jin P, Wang S, Kitamura Y, Yan J, Gao GF 2007 Emergence of fatal PRRSV variants: unparalleled outbreaks of atypical PRRS in China and molecular dissection of the unique hallmark. PLoS One 2:e526. doi:10.1371/journal.pone.0000526. 17565379 PMC1885284

[5] Lager KM, Schlink SN, Brockmeier SL, Miller LC, Henningson JN, Kappes MA, Kehrli ME, Loving CL, Guo B, Swenson SL, Yang HC, Faaberg KS 2014 Efficacy of type 2 PRRSV vaccine against Chinese and Vietnamese HP-PRRSV challenge in pigs. Vaccine 32:6457–6462. doi:10.1016/j.vaccine.2014.09.046. 25285886

[6] Delputte PL, Vanderheijden N, Nauwynck HJ, Pensaert MB 2002 Involvement of the matrix protein in attachment of porcine reproductive and respiratory syndrome virus to a heparinlike receptor on porcine alveolar macrophages. J Virol 76:4312–4320. doi:10.1128/JVI.76.9.4312-4320.2002. 11932397 PMC155060

[7] Van Breedam W, Van Gorp H, Zhang JQ, Crocker PR, Delputte PL, Nauwynck HJ 2010 The M/GP(5) glycoprotein complex of porcine reproductive and respiratory syndrome virus binds the sialoadhesin receptor in a sialic acid-dependent manner. PLoS Pathog 6:e1000730. doi:10.1371/journal.ppat.1000730. 20084110 PMC2799551

[8] Van Breedam W, Delputte PL, Van Gorp H, Misinzo G, Vanderheijden N, Duan X, Nauwynck HJ 2010 Porcine reproductive and respiratory syndrome virus entry into the porcine macrophage. J Gen Virol 91:1659–1667. doi:10.1099/vir.0.020503-0. 20410315

[9] Burkard C, Lillico SG, Reid E, Jackson B, Mileham AJ, Ait-Ali T, Whitelaw CB, Archibald AL 2017 Precision engineering for PRRSV resistance in pigs: macrophages from genome edited pigs lacking CD163 SRCR5 domain are fully resistant to both PRRSV genotypes while maintaining biological function. PLoS Pathog 13:e1006206. doi:10.1371/journal.ppat.1006206. 28231264 PMC5322883

[10] Van Gorp H, Van Breedam W, Van Doorsselaere J, Delputte PL, Nauwynck HJ 2010 Identification of the CD163 protein domains involved in infection of the porcine reproductive and respiratory syndrome virus. J Virol 84:3101–3105. doi:10.1128/JVI.02093-09. 20032174 PMC2826032

[11] Whitworth KM, Rowland RR, Ewen CL, Trible BR, Kerrigan MA, Cino-Ozuna AG, Samuel MS, Lightner JE, McLaren DG, Mileham AJ, Wells KD, Prather RS 2016 Gene-edited pigs are protected from porcine reproductive and respiratory syndrome virus. Nat Biotechnol 34:20–22. doi:10.1038/nbt.3434. 26641533

[12] Pasternak AO, Spaan WJ, Snijder EJ 2006 Nidovirus transcription: how to make sense…? J Gen Virol 87:1403–1421. doi:10.1099/vir.0.81611-0. 16690906

[13] Lee C, Yoo D 2006 The small envelope protein of porcine reproductive and respiratory syndrome virus possesses ion channel protein-like properties. Virology 355:30–43. doi:10.1016/j.virol.2006.07.013. 16904148 PMC7111972

[14] Misinzo GM, Delputte PL, Nauwynck HJ 2008 Involvement of proteases in porcine reproductive and respiratory syndrome virus uncoating upon internalization in primary macrophages. Vet Res 39:55. doi:10.1051/vetres:2008031. 18651989

[15] Guo C, Zhu Z, Guo Y, Wang X, Yu P, Xiao S, Chen Y, Cao Y, Liu X 2017 Heparanase upregulation contributes to porcine reproductive and respiratory syndrome virus release. J Virol 91:e00625-17. doi:10.1128/JVI.00625-17. 28490587 PMC5512235

[16] Sun J, Zhang D, Bae DH, Sahni S, Jansson P, Zheng Y, Zhao Q, Yue F, Zheng M, Kovacevic Z, Richardson DR 2013 Metastasis suppressor, NDRG1, mediates its activity through signaling pathways and molecular motors. Carcinogenesis 34:1943–1954. doi:10.1093/carcin/bgt163. 23671130

[17] Chen B, Nelson DM, Sadovsky Y 2006 N-myc down-regulated gene 1 modulates the response of term human trophoblasts to hypoxic injury. J Biol Chem 281:2764–2772. doi:10.1074/jbc.M507330200. 16314423

[18] van Belzen N, Dinjens WN, Diesveld MP, Groen NA, van der Made AC, Nozawa Y, Vlietstra R, Trapman J, Bosman FT 1997 A novel gene which is up-regulated during colon epithelial cell differentiation and down-regulated in colorectal neoplasms. Lab Invest 77:85–92. 9251681

[19] Piquemal D, Joulia D, Balaguer P, Basset A, Marti J, Commes T 1999 Differential expression of the RTP/Drg1/Ndr1 gene product in proliferating and growth arrested cells. Biochim Biophys Acta 1450:364–373. doi:10.1016/s0167-4889(99)00056-7. 10395947

[20] McCaig C, Potter L, Abramczyk O, Murray JT 2011 Phosphorylation of NDRG1 is temporally and spatially controlled during the cell cycle. Biochem Biophys Res Commun 411:227–234. doi:10.1016/j.bbrc.2011.06.092. 21708134

[21] Kachhap SK, Faith D, Qian DZ, Shabbeer S, Galloway NL, Pili R, Denmeade SR, DeMarzo AM, Carducci MA 2007 The N-Myc down regulated gene1 (NDRG1) is a Rab4a effector involved in vesicular recycling of E-cadherin. PLoS One 2:e844. doi:10.1371/journal.pone.0000844. 17786215 PMC1952073

[22] Chen B, Longtine MS, Sadovsky Y, Nelson DM 2010 Hypoxia downregulates p53 but induces apoptosis and enhances expression of BAD in cultures of human syncytiotrophoblasts. Am J Physiol Cell Physiol 299:C968–C976. doi:10.1152/ajpcell.00154.2010. 20810912 PMC2980304

[23] Liu W, Iiizumi-Gairani M, Okuda H, Kobayashi A, Watabe M, Pai SK, Pandey PR, Xing F, Fukuda K, Modur V, Hirota S, Suzuki K, Chiba T, Endo M, Sugai T, Watabe K 2011 KAI1 gene is engaged in NDRG1 gene-mediated metastasis suppression through the ATF3-NFkappaB complex in human prostate cancer. J Biol Chem 286:18949–18959. doi:10.1074/jbc.M111.232637. 21454613 PMC3099710

[24] Chen Z, Zhang D, Yue F, Zheng M, Kovacevic Z, Richardson DR 2012 The iron chelators Dp44mT and DFO inhibit TGF-beta-induced epithelial-mesenchymal transition via up-regulation of N-Myc downstream-regulated gene 1 (NDRG1). J Biol Chem 287:17016–17028. doi:10.1074/jbc.M112.350470. 22453918 PMC3366822

[25] Liu W, Xing F, Iiizumi-Gairani M, Okuda H, Watabe M, Pai SK, Pandey PR, Hirota S, Kobayashi A, Mo YY, Fukuda K, Li Y, Watabe K 2012 N-myc downstream regulated gene 1 modulates Wnt-beta-catenin signalling and pleiotropically suppresses metastasis. EMBO Mol Med 4:93–108. doi:10.1002/emmm.201100190. 22246988 PMC3306556

[26] Kovacevic Z, Chikhani S, Lui GY, Sivagurunathan S, Richardson DR 2013 The iron-regulated metastasis suppressor NDRG1 targets NEDD4L, PTEN, and SMAD4 and inhibits the PI3K and Ras signaling pathways. Antioxid Redox Signal 18:874–887. doi:10.1089/ars.2011.4273. 22462691

[27] Han B, Li W, Sun Y, Zhou L, Xu Y, Zhao X 2014 A prolyl-hydroxylase inhibitor, ethyl-3,4-dihydroxybenzoate, induces cell autophagy and apoptosis in esophageal squamous cell carcinoma cells via up-regulation of BNIP3 and N-myc downstream-regulated gene-1. PLoS One 9:e107204. doi:10.1371/journal.pone.0107204. 25232961 PMC4169646

[28] Sahni S, Bae DH, Lane DJ, Kovacevic Z, Kalinowski DS, Jansson PJ, Richardson DR 2014 The metastasis suppressor, N-myc downstream-regulated gene 1 (NDRG1), inhibits stress-induced autophagy in cancer cells. J Biol Chem 289:9692–9709. doi:10.1074/jbc.M113.529511. 24532803 PMC3975018

[29] Schweitzer CJ, Zhang F, Boyer A, Valdez K, Cam M, Liang TJ 2018 N-Myc downstream-regulated gene 1 restricts hepatitis C virus propagation by regulating lipid droplet biogenesis and viral assembly. J Virol 92:e01166-17. doi:10.1128/JVI.01166-17. 29118118 PMC5752935

[30] Chen L, Xing C, Ma G, Luo J, Su W, Li M, Shi Q, He H 2018 N-myc downstream-regulated gene 1 facilitates influenza A virus replication by suppressing canonical NF-kappaB signaling. Virus Res 252:22–28. doi:10.1016/j.virusres.2018.05.001. 29730307

[31] Mizushima N, Komatsu M 2011 Autophagy: renovation of cells and tissues. Cell 147:728–741. doi:10.1016/j.cell.2011.10.026. 22078875

[32] Singh R, Cuervo AM 2011 Autophagy in the cellular energetic balance. Cell Metab 13:495–504. doi:10.1016/j.cmet.2011.04.004. 21531332 PMC3099265

[33] Kory N, Farese RV Jr, Walther TC 2016 Targeting fat: mechanisms of protein localization to lipid droplets. Trends Cell Biol 26:535–546. doi:10.1016/j.tcb.2016.02.007. 26995697 PMC4976449

[34] Walther TC, Farese RV Jr 2012 Lipid droplets and cellular lipid metabolism. Annu Rev Biochem 81:687–714. doi:10.1146/annurev-biochem-061009-102430. 22524315 PMC3767414

[35] Singh R, Cuervo AM 2012 Lipophagy: connecting autophagy and lipid metabolism. Int J Cell Biol 2012:282041. doi:10.1155/2012/282041. 22536247 PMC3320019

[36] van Zutphen T, Todde V, de Boer R, Kreim M, Hofbauer HF, Wolinski H, Veenhuis M, van der Klei IJ, Kohlwein SD 2014 Lipid droplet autophagy in the yeast Saccharomyces cerevisiae. Mol Biol Cell 25:290–301. doi:10.1091/mbc.E13-08-0448. 24258026 PMC3890349

[37] Heaton NS, Randall G 2010 Dengue virus-induced autophagy regulates lipid metabolism. Cell Host Microbe 8:422–432. doi:10.1016/j.chom.2010.10.006. 21075353 PMC3026642

[38] Liang XH, Kleeman LK, Jiang HH, Gordon G, Goldman JE, Berry G, Herman B, Levine B 1998 Protection against fatal Sindbis virus encephalitis by Beclin, a novel Bcl-2-interacting protein. J Virol 72:8586–8596. 9765397 10.1128/jvi.72.11.8586-8596.1998 PMC110269

[39] Orvedahl A, Alexander D, Talloczy Z, Sun Q, Wei Y, Zhang W, Burns D, Leib DA, Levine B 2007 HSV-1 ICP34.5 confers neurovirulence by targeting the Beclin 1 autophagy protein. Cell Host Microbe 1:23–35. doi:10.1016/j.chom.2006.12.001. 18005679

[40] Shelly S, Lukinova N, Bambina S, Berman A, Cherry S 2009 Autophagy is an essential component of Drosophila immunity against vesicular stomatitis virus. Immunity 30:588–598. doi:10.1016/j.immuni.2009.02.009. 19362021 PMC2754303

[41] Deretic V, Levine B 2009 Autophagy, immunity, and microbial adaptations. Cell Host Microbe 5:527–549. doi:10.1016/j.chom.2009.05.016. 19527881 PMC2720763

[42] Taylor MP, Kirkegaard K 2007 Modification of cellular autophagy protein LC3 by poliovirus. J Virol 81:12543–12553. doi:10.1128/JVI.00755-07. 17804493 PMC2169029

[43] Dreux M, Gastaminza P, Wieland SF, Chisari FV 2009 The autophagy machinery is required to initiate hepatitis C virus replication. Proc Natl Acad Sci U S A 106:14046–14051. doi:10.1073/pnas.0907344106. 19666601 PMC2729017

[44] Wong J, Zhang J, Si X, Gao G, Mao I, McManus BM, Luo H 2008 Autophagosome supports coxsackievirus B3 replication in host cells. J Virol 82:9143–9153. doi:10.1128/JVI.00641-08. 18596087 PMC2546883

[45] Jackson WT, Giddings TH Jr, Taylor MP, Mulinyawe S, Rabinovitch M, Kopito RR, Kirkegaard K 2005 Subversion of cellular autophagosomal machinery by RNA viruses. PLoS Biol 3:e156. doi:10.1371/journal.pbio.0030156. 15884975 PMC1084330

[46] Sun MX, Huang L, Wang R, Yu YL, Li C, Li PP, Hu XC, Hao HP, Ishag HA, Mao X 2012 Porcine reproductive and respiratory syndrome virus induces autophagy to promote virus replication. Autophagy 8:1434–1447. doi:10.4161/auto.21159. 22739997

[47] Liu Q, Qin Y, Zhou L, Kou Q, Guo X, Ge X, Yang H, Hu H 2012 Autophagy sustains the replication of porcine reproductive and respiratory virus in host cells. Virology 429:136–147. doi:10.1016/j.virol.2012.03.022. 22564420 PMC7111961

[48] Zhou A, Li S, Khan FA, Zhang S 2016 Autophagy postpones apoptotic cell death in PRRSV infection through Bad-Beclin1 interaction. Virulence 7:98–109. doi:10.1080/21505594.2015.1131381. 26670824 PMC4994821

[49] Ellen TP, Ke Q, Zhang P, Costa M 2008 NDRG1, a growth and cancer related gene: regulation of gene expression and function in normal and disease states. Carcinogenesis 29:2–8. doi:10.1093/carcin/bgm200. 17916902

[50] Kovacevic Z, Richardson DR 2006 The metastasis suppressor, Ndrg-1: a new ally in the fight against cancer. Carcinogenesis 27:2355–2366. doi:10.1093/carcin/bgl146. 16920733

[51] He C, Klionsky DJ 2009 Regulation mechanisms and signaling pathways of autophagy. Annu Rev Genet 43:67–93. doi:10.1146/annurev-genet-102808-114910. 19653858 PMC2831538

[52] Cingolani F, Czaja MJ 2016 Regulation and functions of autophagic lipolysis. Trends Endocrinol Metab 27:696–705. doi:10.1016/j.tem.2016.06.003. 27365163 PMC5035575

[53] Singh R, Kaushik S, Wang Y, Xiang Y, Novak I, Komatsu M, Tanaka K, Cuervo AM, Czaja MJ 2009 Autophagy regulates lipid metabolism. Nature 458:1131–1135. doi:10.1038/nature07976. 19339967 PMC2676208

[54] Yang L, Li P, Fu S, Calay ES, Hotamisligil GS 2010 Defective hepatic autophagy in obesity promotes ER stress and causes insulin resistance. Cell Metab 11:467–478. doi:10.1016/j.cmet.2010.04.005. 20519119 PMC2881480

[55] Schroeder B, Schulze RJ, Weller SG, Sletten AC, Casey CA, McNiven MA 2015 The small GTPase Rab7 as a central regulator of hepatocellular lipophagy. Hepatology 61:1896–1907. doi:10.1002/hep.27667. 25565581 PMC4441591

[56] Kimura S, Noda T, Yoshimori T 2007 Dissection of the autophagosome maturation process by a novel reporter protein, tandem fluorescent-tagged LC3. Autophagy 3:452–460. doi:10.4161/auto.4451. 17534139

[57] Pietiainen V, Vassilev B, Blom T, Wang W, Nelson J, Bittman R, Back N, Zelcer N, Ikonen E 2013 NDRG1 functions in LDL receptor trafficking by regulating endosomal recycling and degradation. J Cell Sci 126:3961–3971. doi:10.1242/jcs.128132. 23813961

[58] Reddy JV, Ganley IG, Pfeffer SR 2006 Clues to neuro-degeneration in Niemann-Pick type C disease from global gene expression profiling. PLoS One 1:e19. doi:10.1371/journal.pone.0000019. 17183645 PMC1762405

[59] Bartz F, Kern L, Erz D, Zhu M, Gilbert D, Meinhof T, Wirkner U, Erfle H, Muckenthaler M, Pepperkok R, Runz H 2009 Identification of cholesterol-regulating genes by targeted RNAi screening. Cell Metab 10:63–75. doi:10.1016/j.cmet.2009.05.009. 19583955

[60] Zhou D, Salnikow K, Costa M 1998 Cap43, a novel gene specifically induced by Ni2+ compounds. Cancer Res 58:2182–2189. 9605764

[61] Li J, Liu Y, Wang Z, Liu K, Wang Y, Liu J, Ding H, Yuan Z 2011 Subversion of cellular autophagy machinery by hepatitis B virus for viral envelopment. J Virol 85:6319–6333. doi:10.1128/JVI.02627-10. 21507968 PMC3126540

[62] Sir D, Chen WL, Choi J, Wakita T, Yen TS, Ou JH 2008 Induction of incomplete autophagic response by hepatitis C virus via the unfolded protein response. Hepatology 48:1054–1061. doi:10.1002/hep.22464. 18688877 PMC2562598

[63] Hourioux C, Ait-Goughoulte M, Patient R, Fouquenet D, Arcanger-Doudet F, Brand D, Martin A, Roingeard P 2007 Core protein domains involved in hepatitis C virus-like particle assembly and budding at the endoplasmic reticulum membrane. Cell Microbiol 9:1014–1027. doi:10.1111/j.1462-5822.2006.00848.x. 17257269 PMC2216084

[64] Trask SD, McDonald SM, Patton JT 2012 Structural insights into the coupling of virion assembly and rotavirus replication. Nat Rev Microbiol 10:165–177. doi:10.1038/nrmicro2673. 22266782 PMC3771686

[65] Samsa MM, Mondotte JA, Iglesias NG, Assuncao-Miranda I, Barbosa-Lima G, Da Poian AT, Bozza PT, Gamarnik AV 2009 Dengue virus capsid protein usurps lipid droplets for viral particle formation. PLoS Pathog 5:e1000632. doi:10.1371/journal.ppat.1000632. 19851456 PMC2760139

[66] Guo R, Katz BB, Tomich JM, Gallagher T, Fang Y 2016 Porcine reproductive and respiratory syndrome virus utilizes nanotubes for intercellular spread. J Virol 90:5163–5175. doi:10.1128/JVI.00036-16. 26984724 PMC4859731

[67] Dong H, Czaja MJ 2011 Regulation of lipid droplets by autophagy. Trends Endocrinol Metab 22:234–240. doi:10.1016/j.tem.2011.02.003. 21419642 PMC3118855

[68] Zhang X, Evans TD, Jeong SJ, Razani B 2018 Classical and alternative roles for autophagy in lipid metabolism. Curr Opin Lipidol 29:203–211. doi:10.1097/MOL.0000000000000509. 29601311 PMC5930069

[69] Singh R, Xiang Y, Wang Y, Baikati K, Cuervo AM, Luu YK, Tang Y, Pessin JE, Schwartz GJ, Czaja MJ 2009 Autophagy regulates adipose mass and differentiation in mice. J Clin Invest 119:3329–3339. doi:10.1172/JCI39228. 19855132 PMC2769174

[70] Wensvoort G, Terpstra C, Pol JM, ter Laak EA, Bloemraad M, de Kluyver EP, Kragten C, van Buiten L, den Besten A, Wagenaar F, Broekhuijsen JM, Moonen PLJM, Zetstra T, de Boer EA, Tibben HJ, de Jong MV, van’t Veld P, Greenland GJR, van Gennep JA, Voets MT, Verheijden JHM, Braamskamp J 1991 Mystery swine disease in The Netherlands: the isolation of Lelystad virus. Vet Q 13:121–130. doi:10.1080/01652176.1991.9694296. 1835211

[71] Wang C, Huang B, Kong N, Li Q, Ma Y, Li Z, Gao J, Zhang C, Wang X, Liang C, Dang L, Xiao S, Mu Y, Zhao Q, Sun Y, Almazan F, Enjuanes L, Zhou EM 2013 A novel porcine reproductive and respiratory syndrome virus vector system that stably expresses enhanced green fluorescent protein as a separate transcription unit. Vet Res 44:104. doi:10.1186/1297-9716-44-104. 24176053 PMC4176086

[72] Ma H, Jiang L, Qiao S, Zhi Y, Chen XX, Yang Y, Huang X, Huang M, Li R, Zhang GP 2017 The crystal structure of the fifth scavenger receptor cysteine-rich domain of porcine CD163 reveals an important residue involved in porcine reproductive and respiratory syndrome virus infection. J Virol 91:e01897-16. doi:10.1128/JVI.01897-16. 27881657 PMC5244331

[73] Choi YS, Lee JK, Jung JT, Jung YC, Jung JH, Jung MO, Choi YI, Jin SK, Choi JS 2016 Comparison of meat quality and fatty acid composition of longissimus muscles from purebred pigs and three-way crossbred LYD pigs. Korean J Food Sci Anim Resour 36:689–696. doi:10.5851/kosfa.2016.36.5.689. 27857546 PMC5112433

[74] Wang J, Chu B, Du L, Han Y, Zhang X, Fan S, Wang Y, Yang G 2015 Molecular cloning and functional characterization of porcine cyclic GMP-AMP synthase. Mol Immunol 65:436–445. doi:10.1016/j.molimm.2015.02.002. 25765883

[75] Reed LJ, Muench H 1938 A simple method of estimating fifty per cent endpoints. Am J Hyg 27:493–497. doi:10.1093/oxfordjournals.aje.a118408.

